# Bedmap3 updated ice bed, surface and thickness gridded datasets for Antarctica

**DOI:** 10.1038/s41597-025-04672-y

**Published:** 2025-03-10

**Authors:** Hamish D. Pritchard, Peter T. Fretwell, Alice C. Fremand, Julien A. Bodart, James D. Kirkham, Alan Aitken, Jonathan Bamber, Robin Bell, Cesidio Bianchi, Robert G. Bingham, Donald D. Blankenship, Gino Casassa, Knut Christianson, Howard Conway, Hugh F. J. Corr, Xiangbin Cui, Detlef Damaske, Volkmar Damm, Boris Dorschel, Reinhard Drews, Graeme Eagles, Olaf Eisen, Hannes Eisermann, Fausto Ferraccioli, Elena Field, René Forsberg, Steven Franke, Vikram Goel, Siva Prasad Gogineni, Jamin Greenbaum, Benjamin Hills, Richard C. A. Hindmarsh, Andrew O. Hoffman, Nicholas Holschuh, John W. Holt, Angelika Humbert, Robert W. Jacobel, Daniela Jansen, Adrian Jenkins, Wilfried Jokat, Lenneke Jong, Tom A. Jordan, Edward C. King, Jack Kohler, William Krabill, Joséphine Maton, Mette Kusk Gillespie, Kirsty Langley, Joohan Lee, German Leitchenkov, Cartlon Leuschen, Bruce Luyendyk, Joseph A. MacGregor, Emma MacKie, Geir Moholdt, Kenichi Matsuoka, Mathieu Morlighem, Jérémie Mouginot, Frank O. Nitsche, Ole A. Nost, John Paden, Frank Pattyn, Sergey Popov, Eric Rignot, David M. Rippin, Andrés Rivera, Jason L. Roberts, Neil Ross, Antonia Ruppel, Dustin M. Schroeder, Martin J. Siegert, Andrew M. Smith, Daniel Steinhage, Michael Studinger, Bo Sun, Ignazio Tabacco, Kirsty J. Tinto, Stefano Urbini, David G. Vaughan, Douglas S. Wilson, Duncan A. Young, Achille Zirizzotti

**Affiliations:** 1 British Antarctic Survey, Cambridge, UK; 2 Climate and Environmental Physics, University of Bern, Bern, Switzerland; 3 Oeschger Centre for Climate Change Research, University of Bern, Bern, Switzerland; 4 School of Earth and Oceans and the Australian Centre for Excellence in Antarctic Science, The University of Western Australia, Perth, Western Australia Australia; 5 School of Geographical Sciences, University of Bristol, Bristol, UK; 6 Department of Aerospace and Geodesy, Technical University of Munich, Munich, Germany; 7 Lamont-Doherty Earth Observatory of Columbia University, Palisades, USA; 8 Istituto Nazionale di Geofisica e Vulcanologia, Rome, Italy; 9 School of GeoSciences, University of Edinburgh, Edinburgh, UK; 10 Institute for Geophysics, University of Texas at Austin, Austin, USA; 11 General Directorate of Water (DGA), Santiago, Chile; 12 University of Magallanes, Punta Arenas, Chile; 13 Earth and Space Sciences, University of Washington, Seattle, USA; 14 Polar Research Institute of China, Shanghai, China; 15 Federal Institute for Geosciences and Natural Resources, Hanover, Germany; 16 Alfred Wegener Institute Helmholtz Centre for Polar and Marine Research, Bremerhaven, Germany; 17 Department of Geosciences, Tübingen University, Tübingen, Germany; 18 Department of Geoscience, University of Bremen, Bremen, Germany; 19 Istituto Nazionale di Oceanografia e di Geofisica Sperimentale, Trieste, Italy; 20 DTU Space, Lyngby, Denmark; 21 National Centre for Polar & Ocean Research (NCPOR), Ministry of Earth Sciences, Vasco-da Gama, Goa, India; 22 University of Alabama, Tuscaloosa, AL 35487 USA; 23 Scripps Institution of Oceanography, University of California in San Diego, La Jolla, USA; 24 Colorado School of Mines, Department of Geophysics, Golden, CO USA; 25 Amherst College, Amherst, USA; 26 Lunar and Planetary Laboratory, University of Arizona, Tucson, USA; 27 St. Olaf College, Northfield, MN 55057 USA; 28 Department of Geography and Environmental Sciences, Northumbria University, Newcastle upon Tyne, UK; 29 Australian Antarctic Division, Kingston, Australia; 30 Australian Antarctic Program Partnership, Institute for Marine & Antarctic Studies, University of Tasmania, Hobart, Australia; 31 Norwegian Polar Institute, Fram Centre, Tromsø, Norway; 32 NASA Wallops Flight Facility, Wallops Island, VA USA; 33 Norwegian Polar Institute, Tromso, Norway; 34 Western Norway University of Applied Sciences, Sogndal, Norway; 35 Asiaq, Greenland Survey, Nuuk, Greenland; 36 Korea Polar Research Institute, Incheon, South Korea; 37 Institute for Geology and Mineral Resources of the World Ocean, St. Petersburg, Russia; 38 Saint Petersburg State University, Petersburg, Russia; 39 Centre for Remote Sensing and Integrated Systems, University of Kansas, Lawrence, USA; 40 Earth Research Institute, University of California in Santa Barbara, Santa Barbara, USA; 41 Cryospheric Sciences Lab, NASA Goddard Space Flight Center, Greenbelt, Maryland USA; 42 Department of Geological Sciences, University of Florida, Gainesville, USA; 43 Department of Earth Sciences, Dartmouth College, Hanover, USA; 44 Department of Earth System Science, University of California Irvine, Irvine, CA USA; 45 University of Grenoble Alpes, CNRS, IRD, Grenoble INP, IGE, Grenoble, France; 46 Oceanbox.io, Tromsø, Norway; 47 Laboratoire de Glaciologie, Université Libre de Bruxelles, Brussels, Belgium; 48 Polar Marine Geosurvey Expedition, St. Petersburg, Russia; 49 Department of Civil and Environmental Engineering, University of California Irvine, Irvine, CA USA; 50 Jet Propulsion Laboratory, California Institute of Technology, Pasadena, USA; 51 Department of Environment and Geography, University of York, York, UK; 52 Departamento de Geografía, Universidad de Chile, Santiago, Chile; 53 School of Geography, Politics and Sociology, Newcastle University, Newcastle upon Tyne, UK; 54 Department of Geophysics, Stanford University, Stanford, CA USA; 55 Department of Electrical Engineering, Stanford University, Stanford, CA USA; 56 University of Exeter, Penryn Campus, Penryn, Cornwall, UK; 57 NASA Goddard Space Flight Center, Greenbelt, USA; 58 Marine Science Institute, University of California Santa Barbara, Santa Barbara, USA

**Keywords:** Cryospheric science, Hydrology, Cryospheric science, Geomorphology

## Abstract

We present Bedmap3, the latest suite of gridded products describing surface elevation, ice-thickness and the seafloor and subglacial bed elevation of the Antarctic south of 60 °S. Bedmap3 incorporates and adds to all post-1950s datasets previously used for Bedmap2, including 84 new aero-geophysical surveys by 15 data providers, an additional 52 million data points and 1.9 million line-kilometres of measurement. These efforts have filled notable gaps including in major mountain ranges and the deep interior of East Antarctica, along West Antarctic coastlines and on the Antarctic Peninsula. Our new Bedmap3/RINGS grounding line similarly consolidates multiple recent mappings into a single, spatially coherent feature. Combined with updated maps of surface topography, ice shelf thickness, rock outcrops and bathymetry, Bedmap3 reveals in much greater detail the subglacial landscape and distribution of Antarctica’s ice, providing new opportunities to interpret continental-scale landscape evolution and to model the past and future evolution of the Antarctic ice sheets.

## Background & Summary

The Bedmap products have been key inputs for models of past, present and future ice behaviour, and for identifying areas of the ice sheet that are potentially vulnerable to collapse (e.g., refs. ^1–7^). A decade on from the release of Bedmap2 (ref. ^8^) and two decades since Bedmap1 (ref. ^9^), the need for accurate and detailed, consistent, continent-wide mapping of Antarctic surface topography, ice thickness and bed topography has never been greater. The response of the Antarctic Ice Sheet to climate change remains the greatest source of uncertainty in the rate of sea level rise over the next few decades and beyond^10^. It is these parameters, the fundamental controls on ice dynamics and also key determinants of ice-ocean interaction, that lie at the heart of the urgent challenge to model future rates of ice loss^11,12^. Furthermore, Antarctica’s subglacial landscape records direct evidence of the continent’s tectonic and geomorphological history (e.g.^13,14^), and along with ice thickness, is a primary control on the flow of water and the distribution of lacustrine habitats under the ice (e.g.^15,16^). The value and wide range of uses for the Bedmap products are evidenced by around 130 scientific citations per year to date, but as new survey datasets and mappings of Antarctic surface topography, bathymetry, rock outcrops and the grounding line continue to become available, an update to these products is needed.

**Fig. 1:**
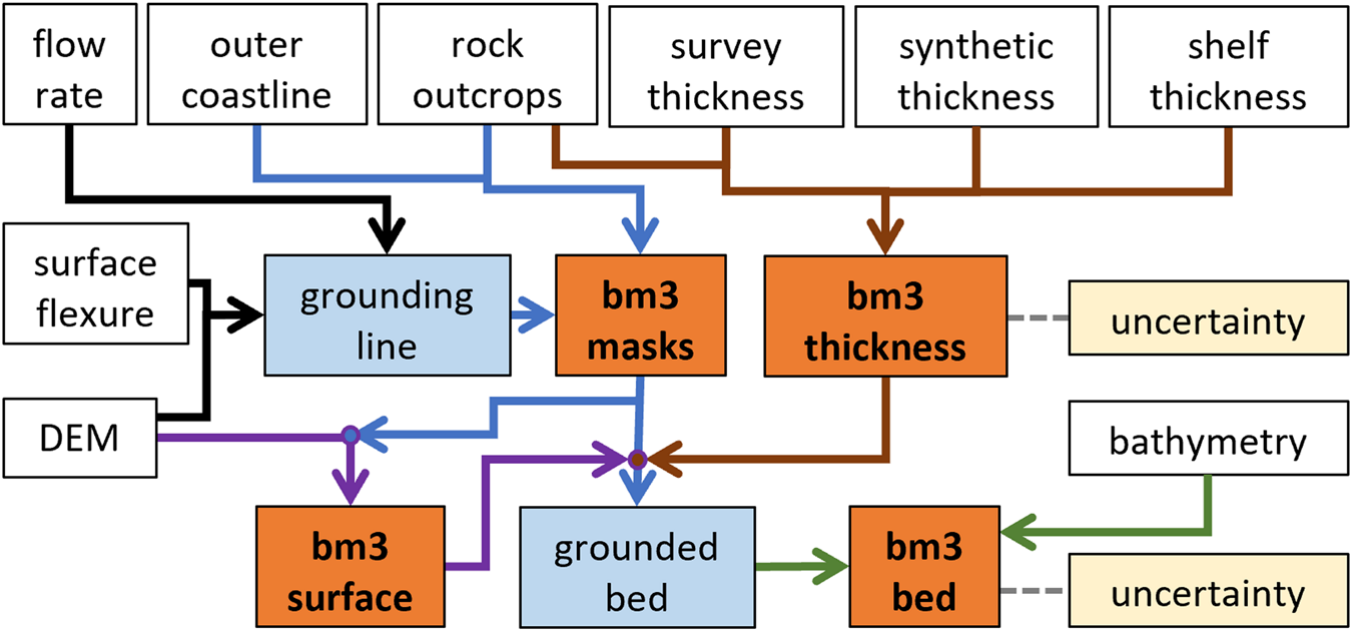
Schematic showing the Bedmp3 source datasets (white boxes) combining to make intermediate products (blue boxes) and ultimately the final set of grids (orange boxes) and their uncertainties (yellow boxes). Note that the surface grid has a uniform estimated uncertainty (Section *Uncertainty estimates*).

**Fig. 2:**
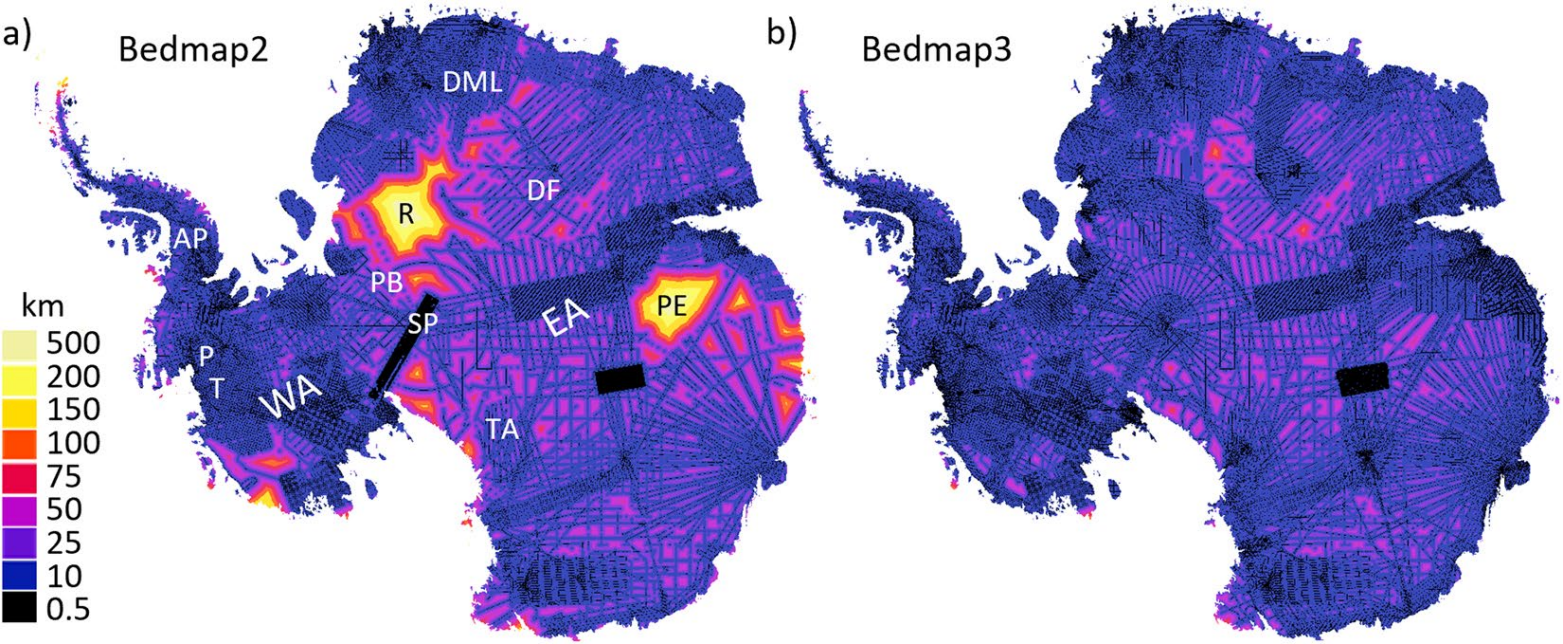
The survey coverage of Antarctica has improved since Bedmap2. This has decreased the distance from interpolated cells to ice thickness survey data (black lines) from that of (**a**) Bedmap2 (ref. ^8^) to that of (**b**) Bedmap3. P = Pine Island Glacier, T = Thwaites Glacier, R = Recovery Glacier, PE = Princess Elizabeth Land, SP = South Pole, PB = Pensacola Basin, DML = Dronning Maud Land, AP = Antarctic Peninsula, WA = West Antarctica, EA = East Antarctica, TA = Transantarctic Mountains, DF = Dome Fuji.

Here we report on Bedmap3, the latest continent-wide mapping of Antarctic surface topography, ice thickness, bed topography and masks defining the distribution of the grounded ice sheet, ice shelves and rock (Fig. 1). This iteration makes use of all currently available ice-thickness survey datasets, with 84 new aero-geophysical surveys of ice thickness since Bedmap2 (an additional 52 million data points and 1.9 million line-kilometres of measurement) that have filled notable gaps in East Antarctica, including the South Pole and Pensacola basin, Dronning Maud Land, Recovery Glacier and Dome Fuji, Princess Elizabeth Land, plus the Antarctic Peninsula, West Antarctic coastlines, and the Transantarctic Mountains^17^ (Fig. 2). In Bedmap3, the mean distance between a grid cell and an ice thickness measurement is 5.6 km (Standard Deviation (SD) 7.3 km) and the maximum is 98 km, less than half that of Bedmap2 (Fig. 2).

For Bedmap3 we employed substantially the same methods as Bedmap2 (ref. ^8^) to interpolate and combine these survey data with multiple other input datasets (including bathymetry, surface topography, rock outcrops, grounding line location, coastline, and derived ice shelf thicknesses (Fig. 1)), with the caveat that some steps (described in the Methods) required specific judgments to be made regarding conflicting measurements: hence not all measurements are honoured. We took care to ensure self-consistency between the grids of ice surface elevation, bed-elevation and ice thickness and the known status of grid cells as either floating or grounded ice, and we endeavoured to avoid interpolation artefacts such as abrupt steps when combining datasets across important physical boundaries such as the grounding line. Our overarching aim remained to produce a complete, consistent gridded product that can resolve features important to modelling the grounding zones of ice streams, for example, but remains appropriate for use in a wide range of scientific disciplines. Bedmap3 therefore remains data-driven and, as for previous Bedmaps, we avoid the use of ice sheet models or the inversion of bed topography from surface properties (e.g.^18–27^) that offer significant opportunities to refine bed and thickness mapping for certain applications but require model-specific assumptions.

The resulting Bedmap3 grids represent a comprehensive update to the mapping of Antarctica’s subaerial, subglacial, and submarine landscapes. The derived statistics for Bedmap3 show that the volume of ice contained in the Antarctic ice sheet (27.17 million km^3^) and its potential contribution to sea-level rise (58 m) are similar to those of Bedmaps 1 and 2 (Section *Comparison to earlier Bedmap products and BedMachine v3*), but the topography of these hidden landscapes is revealed in unprecedented detail in many areas (Section *Bed topography*).

## Methods

We created the Bedmap3 classification mask and grids of surface elevation, ice thickness, bed topography and uncertainty from a combination of new and existing interpolations of discontinuous survey datasets (airborne and ground-survey ice thickness, ship-borne bathymetry), existing gridded datasets from satellite-based mapping of rock outcrops, surface topography and ice shelf thickness, and a compilation of grounding zone coastal features extracted from satellite analysis. Here we describe the data sources, the methods used to create the new grids, and the output products. These outputs were created in the ArcGIS Pro 3.3.1 software package.

### Geoid, projection, and grid cell size

For Bedmap3 we employ the same geoid (gl04c) and projection (WGS 1984 Antarctic Polar Stereographic: EPSG:3031) as for Bedmap2, with projected extents of: top 3,333,500 m, bottom -3,333,500 m, left -3,333,500 m and right 3,333,500 m. The publication of all input ice thickness survey data^17^ allows us to grid at 500 m spacing, a high resolution that is increasingly demanded by the ice and ocean modelling communities for simulating grounding line movement and thus ice sheet stability^12,28,29^. This contrasts with the Bedmap2 products that were interpolated to a native 5 km grid then oversampled to 1 km, as a condition of use by the data contributors at that time. The finer 500 m gridding in Bedmap3 helps to resolve in detail features such as subglacial trough cross-profiles where measurements are distributed relatively densely along survey lines. In almost all cases, however, the spatial distribution of the survey data remains highly anisotropic: along-track sampling is universally denser (typically metres to kilometres) than the sparse across-track sampling (typically hundreds of metres to hundreds of km). As a result, 93% of the 500 m Bedmap3 grid cells of ice thickness are populated by interpolated values (Fig. 2, Section *Interpolation of the ice thickness grid*).

### Masks of grounded ice, transient grounded ice, floating ice shelf and rock

In collaboration with the Scientific Committee on Antarctic Research (SCAR) Action Group Antarctic RINGS (https://scar.org/science/cross/rings), we have produced a revised definition of the limits of floating and grounded ice for Antarctica which have evolved in the decade since Bedmap2 was published. Grounding zone properties (such as the landward and seaward limits of tidal flexure and the visible surface break in slope associated with the grounded-floating transition) have previously been mapped with a variety of methods (laser and radar altimetry, InSAR, optical image processing) over various spatial scales, time periods and sampling frequencies. Most are spatially discontinuous, and all represent disparate snapshots of the grounding line location as it oscillates through tidal cycles and, in some cases, progressively or abruptly migrates over time. Some of these mappings are mutually inconsistent, some contain obvious errors and there is no continuous, recent and universally accepted grounding line for the whole continent (e.g.^30^).

**Fig. 3:**
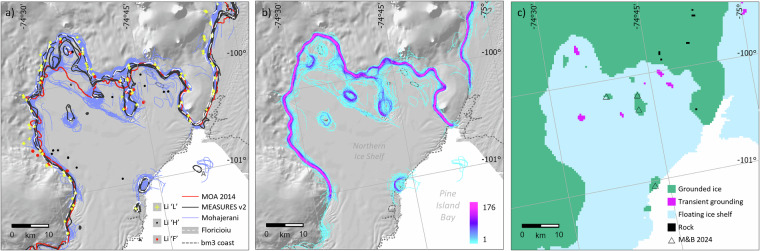
The new Bedmap3 grounding line seeks to reconcile multiple varying observations. (**a**) A suite of published grounding lines and associated tidal flexure limits on the Northern Ice Shelf of Pine Island Glacier mapped by InSAR, ICESat, Cryosat2, MODIS and Landsat (Table 1) (background image: shaded relief of surface elevation after ref. ^41^). Note that feature ‘A’ is no longer covered by the ice shelf; (**b**) a heatmap of grounding line location from multiple lines mapped by InSAR through 2018 (after ref. ^35^); (**c**) the reconciled Bedmap3 masks of grounded ice, ‘transient grounding’, floating ice shelf and rock, gridded at 500 m. For comparison, M&B 2024 refers to pinning points identified in a separate study^44^.

To produce an updated grounding line, we referred to several InSAR-derived^31–35^, altimetry-derived^36,37^, imagery-derived^38,39^ and multi-source^40^ grounding line products, plus a shaded-relief version of the REMA digital elevation model^41^ as a visual check on grounding line location (e.g., Fig. 3a). To address the tidal variability of the grounding line location, we generated a heat map of its automatically interpreted location (Fig. 3b) from a densely time-sampled suite of grounding line snapshots, where available^35^. We digitised the modal line of this distribution where it broadly agreed with the other datasets cited above and lay in a physically reasonable location, avoiding obvious errors in the automated mapping (e.g., areas of high ground^41^ or offshore). This modal line represents the average grounding line location through year 2018.

Where these densely sampled snapshots were not available, we digitised the grounding line by linking the discrete altimetry-derived mappings of the associated break in slope that lies between the landward and seaward tidal-flexure limits (points termed *I*_*b*_ in ref. ^37^), or a comparable point between these limits^36^, where available. In other areas, we adopted InSAR-derived mappings for Larsen C Ice Shelf^31^ and Thwaites Glacier^34^ plus, more broadly, two existing Antarctic-wide compilations^33,42^. Having mapped the grounding line for all landward ice-shelf margins, we merged this with the coastline and ice-shelf seaward limits digitised from Landsat 8 images acquired during January-March 2022.

In an improvement to the Bedmap2 masks depicting grounded versus floating ice, we have added another category for ‘transient grounded’ areas of ice shelf, where such features could be identified from the various products described above (Fig. 3c). We identified these as having fewer detected groundings relative to the neighbouring grounding zone with the landward ice sheet, and we verified these manually as having some surface expression in visible satellite imagery, surface topography^41^ and surface velocity^43^, but being less pronounced than for permanently grounded ice rises. These zones of transient grounding, which may influence ice dynamics and sub-shelf ocean circulation, represent areas that ground only at low tidal states or became ungrounded due to ice shelf thinning during the observed period of 1994-2020, with most observations spanning 2015-2020 (Table 1). Our mapping of grounded and transiently grounded features agrees well with and adds to a separate mapping of similar ‘pinning point’ features for selected years up to 2022 (ref. ^44^) (e.g., ‘M&B 2024’ in Fig. 3c). Of the 660 pinning points identified by that study that lie within the overall Bedmap3 mask, 95% coincide with grounded or transiently grounded Bedmap3 features.

**Table 1:** Sources, methods, and date ranges of grounding line products.

Grounding line data source	Primary method	Date range
Christie, *et al*.^31^	InSAR	2019
Floricioiu, *et al*.^32^	InSAR	1994-2020
Rignot, *et al*.^33^	InSAR (MEASURES v2)	2018-2020
Milillo, *et al*.^34^	InSAR	1992-2017
Mohajerani, *et al*.^35^	InSAR	2018
Dawson and Bamber^36^	Radar altimetry	2011-2013
Li, *et al*.^37^	Laser altimetry	2019-2020
Scambos, *et al*.^38^	Optical image analysis (MOA)	2003-2004
Haran, *et al*.^39^	Optical image analysis (MOA 2014)	2008-2009

To complete the Bedmap3 classification mask^45^, we merged the above classes with a rock mask for the continent and surrounding islands modified from the Antarctic Digital Database (ADD) version 7.4 derived from Landsat8 images^46^. We manually edited this rock outcrop map using visible satellite images to remove “rock” areas that actually represented supraglacial medial and lateral moraine that overlie ice, rather than bedrock. The mask classes are: 1 – grounded ice (48,083,577 cells); 2 – transient grounded ice (972 cells); 3 – floating iceshelf (6,153,150 cells); 4 – rock (302,508 cells).

### Ice thickness data and gridding

#### Filtering and weighting of survey data

The 277 ice thickness surveys from the last 60 years employed here collected a total of 82 million ‘raw’ data points which were statistically summarised by individual survey into a set of 4.13 million ‘summarised points’ (summarised by a count of the points, their mean, standard deviation, median, interquartile range etc. for each survey mission) aggregated onto a 500 m grid^17^, of which 3.64 million cover the grounded ice sheet (see Background and Summary). These data vary in sampling rate and reliability. In well-surveyed areas, disagreements in interpreted thicknesses from overlapping missions may reflect the lower accuracy in navigation, lower accuracy in depth sounding from analogue versus digitally recorded radargrams, the choice of bed picking algorithm and technique, and the lower radar pulse repetition frequency and hence the frequency of thickness measurements of older, less sophisticated surveys. As for Bedmap2, in all cases we used the ice thicknesses reported by the survey owners, which are based on their own assessments of the radar velocity in ice.

Where summarised points from multiple surveys were present within a Bedmap3 grid cell, we calculated a weighted mean cell thickness according to the number of measurements made by each survey (using the point count (Cnt_thick) summary statistic^17^). This approach effectively favours more recent, densely sampled surveys, down-weighting (but not eliminating) sparser, older data where present. We also excluded measurements with non-zero thickness over known rock outcrops, or measurements reporting zero thickness over known ice (Section *Masks of grounded ice, transient grounded ice, floating ice shelf and rock*). In data-rich areas where ice thickness is known to have changed through time, notably in the Amundsen Sea Embayment of West Antarctica^11^, we excluded isolated, single-point measurements (Cnt_thick = 1 per cell) because we found that such old, single-shot surveys tended to introduce large anomalies during subsequent thickness interpolation. We also manually excluded some other older observations where they disagreed with more recent surveys.

After being filtered and averaged, the mean survey year for cells on the main lower trunks of Pine Island and Thwaites Glaciers, for example, is 2009.4 and 2010.6 respectively, in contrast with a mean of 2006.9 for the full grounded ice sheet (Table 2). Overall, we filtered out 8% of the original 3.64 million summarised points and from the remaining points, generated 3.15 million weighted-mean cell values.

**Table 2:** Mean years of summarised ice thickness survey points in Bedmap3, for selected areas shown in Fig. 2.

Zone	Summarised points (approx., after filtering)	Mean raw points per grid cell (range)	Mean year (SD)
Pine Island Glacier lower trunk	44,000	35.7 (1-548)	2009.4 (5.4)
Thwaites Glacier lower trunk	67,000	110.4 (1-2034)	2010.6 (5.7)
Recovery Basin	27,000	18.8 (1-347)	2014.2 (1.5)
Princess Elizabeth Land	18,000	20.8 (1-170)	2015.6 (1.2)
Full grounded ice sheet	3,570,000	18.52 (1-4399)	2006.9 (7.6)

Recovery Basin and Princess Elizabeth Land statistics apply to the two labelled areas within the 100 km contour in Fig. 2a.

#### Ice shelf thickness

We employed an updated ice shelf thickness grid relative to that of Bedmap2, derived from CryoSat-2 satellite altimetry of the ice shelf freeboard spanning 2011–2014 (ref. ^47^). This grid provides consistency in data source, processing and measurement epoch, and is based on 92.3% data coverage over the ice shelves, improving upon previous products around the grounding zone^47^. The freeboard-based approach to estimating ice shelf thickness depends upon the shelf being in hydrostatic equilibrium, which may not apply near the grounding line if the shelf is partially laterally supported by coastal land. Where previously tested, thickness biases were in most cases greater within 10 km of the grounding line than elsewhere^47^. To minimise bias introduced by failure of the assumption of hydrostatic equilibrium, and to aid in smooth interpolation of thickness across the grounding zone (Section *Interpolation of the ice thickness grid*), for Bedmap3 we excluded freeboard-derived shelf thicknesses from all areas within 3 km of the grounding line, and those areas that we identified as grounded or transiently grounded (Section *Masks of grounded ice, transient grounded ice, floating ice shelf and rock*). When in doubt, we extended this exclusion zone farther seaward to the limit of hydrostatic equilibrium indicated by grounding-zone mapping^36,37^.

Freeboard-based ice shelf thickness products also require an estimate of the column-averaged shelf density, which varies spatially and is imperfectly known. Shelf density was previously modelled and the resulting total column thicknesses assessed against survey data over a subset of ice shelves, but biases were not systematically corrected for^47^. Employing Bedmap3’s larger compilation of survey data^17^, we calibrated the altimetry-derived shelf thickness grid by quantifying the offset between it and all ice shelf airborne or field survey data (1.08 million raw measurements aggregated to 447,327 summarised points). Importantly, on the Filchner Ronne Ice Shelf (FRIS) and Amery Ice Shelf we excluded radar survey data that coincided with mapped basal marine ice^48–50^, as marine ice is opaque to radar and causes a low bias in the radar-surveyed thicknesses. For the FRIS, we instead tested the gridded thickness using a set of 987 seismic survey thickness points within the Bedmap3 database^17^ which are not biased by the presence of marine ice.

**Fig. 4:**
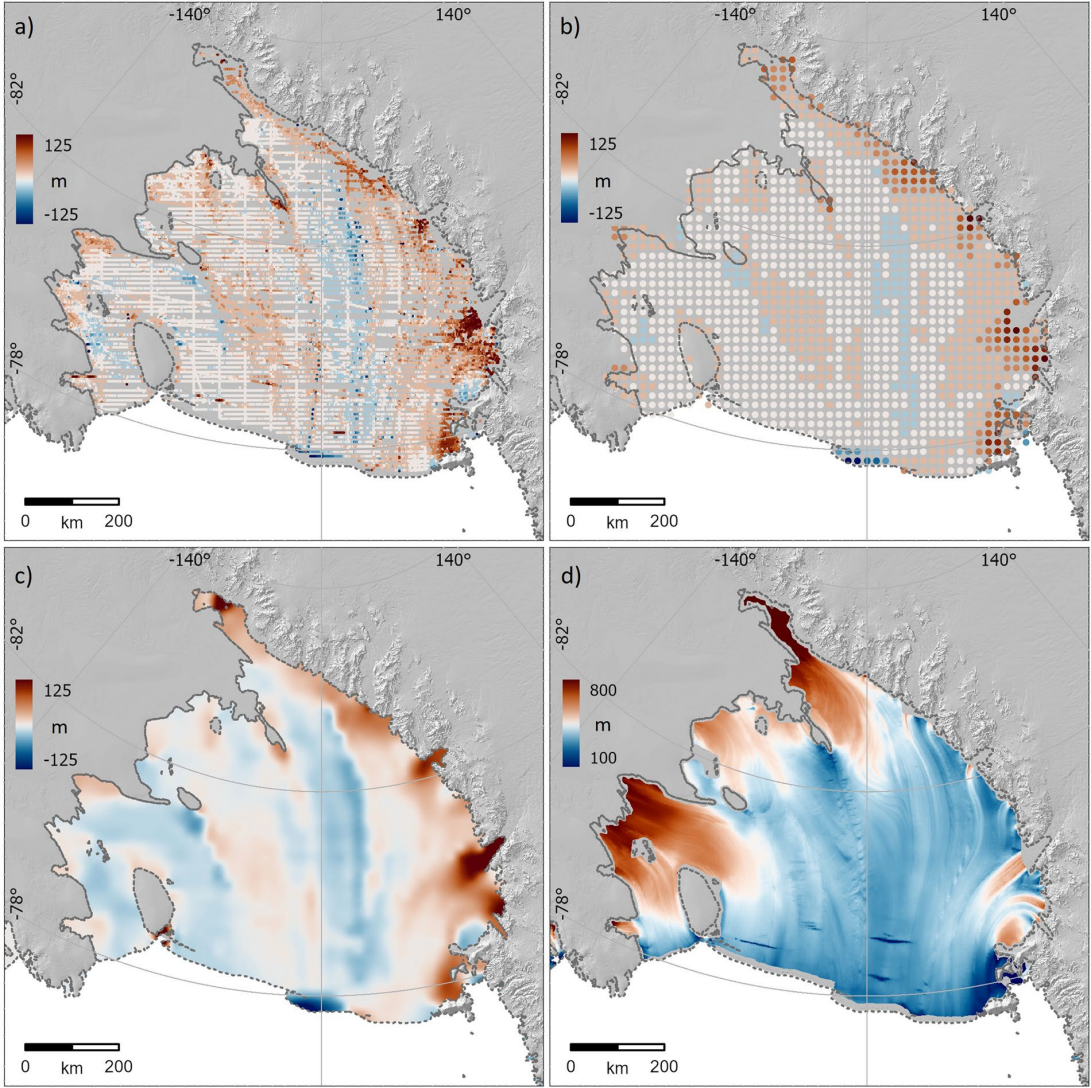
The Bedmap3 survey dataset allows us to calibrate the more continuous and extensive freeboard-based ice shelf thickness grid. (**a**) Pointwise ice thickness biases between 119,000 survey measurements and the freeboard-derived ice thickness grid^47^ on the Ross Ice Shelf, showing coherent patterns of bias; (**b**) these pointwise offsets median-filtered over a 20 km radius; (**c**) spline-interpolated calibration grid of the filtered offsets in (**b**); (**d**) bias-corrected version of the freeboard-derived ice thickness grid after subtraction of the calibration grid in (**c**).

The mean offsets for well-surveyed shelves were typically small (Table 3), but offsets varied spatially and had coherent patterns that precluded a uniform bias correction for the freeboard-derived thickness grid (e.g., Fig. 4a). To correct for the spatially varying biases that we identified, we filtered our pointwise bias measurements using a median filter (radius 20-30 km according to shelf size) (Fig. 4b) and smoothed and interpolated them to the full shelf extent (using the ArcGIS ‘Spline with barriers’ algorithm with smoothing of 0.5). This produced a set of spatially continuous but variable 500 m calibration grids for the ice shelves that we subtracted from the freeboard product (Fig. 4c) to make a calibrated thickness grid (Fig. 4d). After calibration, this new Bedmap3 ice shelf thickness grid has a mean of 468 m (SD 244 m) for all Antarctic ice shelves compared to the original freeboard-based grid^47^ mean of 467 m (SD 245 m).

**Table 3:** Mean biases identified in uncalibrated freeboard-derived shelf thicknesses.

Shelf	Mean bias (m)	SD (m)
Amery	7.3	21.4
Ross	1.3	31.3
FRIS	2.0	53.0
Other shelves	2.1	43.1

#### Synthetic ice thickness data: Glen’s flow law for ‘thin ice’

Ice thickness survey data are particularly scarce close to Antarctica’s rock outcrops (within mountain ranges and around nunataks, isolated knolls and coastal areas), which serve as zones of known zero ice thickness for interpolation to a continuous grid. Because such dense blocks of zero-thickness data can come to dominate interpolation in the absence of similarly dense ice survey data, Bedmap 1 and 2 employed a single, empirically derived relationship between surveyed thicknesses and distance from rock, to enforce positive ice thicknesses for ‘thin ice’ at distances of up to 10 km around rock features.

For Bedmap3, we were able to make use of more recently available, high-resolution maps of surface flow speed^43^ and slope (after^41^), plus an Antarctic-wide map of mean annual air temperature (after^51^) and firn-air-content^18^, to make a locally adaptive estimate of ice thicknesses (*H*) around outcrops using Glen’s flow law (Eq. (1)).

1 $$H={\left\lfloor \frac{{4U}_{s}}{A{\left({\rho }_{c}{gsina}\right)}^{n}}\right\rfloor }^{1/4}$$

As this law applies to creep flow, we limited its application to areas that are both slow-flowing (*U*_*s*_ ≤ 30 ma^−1^) and with relatively steep slopes (α ≥ 2° calculated over a 1500 m distance, several times the expected ice thickness^52^). We further limited it to areas within 15 km of rock or within 20 km of the coastline/grounding line (to capture ice-covered coastal slopes). We used the exponent *n* = 3 (well suited to ice with little-developed fabric and few impurities^52^), and the multi-decadal mean annual surface temperature to estimate the spatially varying temperature-dependent Arrhenius parameter (*A)*, a relationship that is relatively well understood for the predominantly low (<−10 °C) Antarctic temperatures^52^. We used the spatially varying firn-air-content to estimate the column-averaged density (*ρ*_c_) relative to that of ice, allowing us to estimate the total column thickness. Given the uncertainties, particularly in *n* and *A*, we calibrated our calculated (Glen) thicknesses against 84,984 radar-derived survey measurements falling within the ‘thin ice’ domain. For these points, we found the median surveyed thickness (270 m) to be 1.44 times greater than the median calculated thickness (187 m), so we scaled all calculated thicknesses by this factor.

**Fig. 5:**
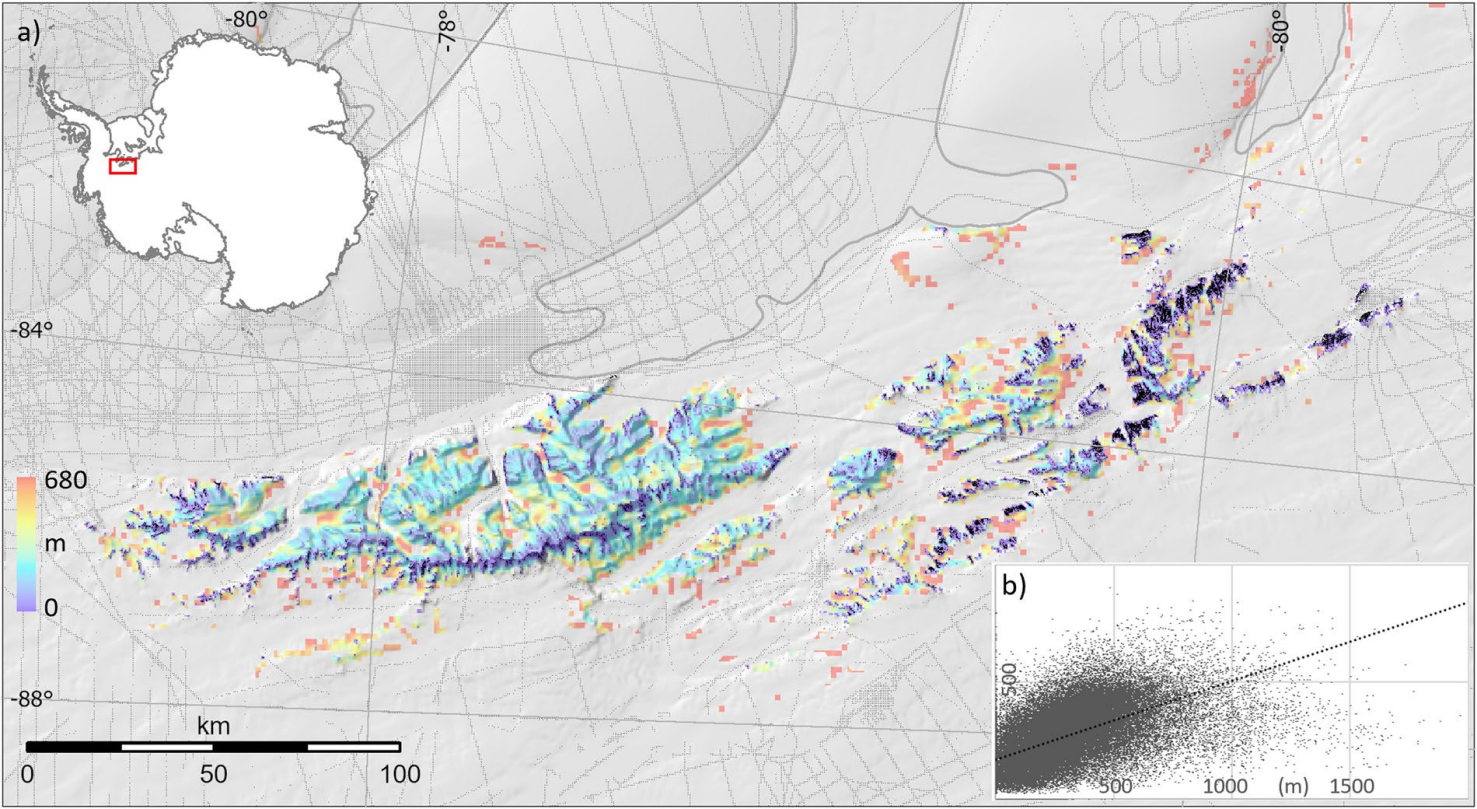
(**a**) Synthetic ice thickness of unsurveyed areas in the Ellsworth mountains (within 15 km of rock or within 20 km of the coastline/grounding line). Thin grey lines show thickness surveys, the thick grey line shows the Bedmap3 grounding line, rock outcrops are shown in black. (**b**) scatterplot showing surveyed thickness (metres, x axis) plotted against calculated ‘Glen’ thicknesses (metres, y axis) and the linear regression line between the two (dashed black line).

Survey data available for such calibration remain limited both in extent and reliability in Antarctica’s mountainous terrain, where radar signals are more difficult to interpret^53^, and their correlation with flow-law-derived thicknesses is relatively low (R^2^ = 0.314, Fig. 5b). However, these calculated thicknesses yield a plausible ice distribution around outcrops (e.g., Fig. 5a) that is determined by the local slope, flow speed and climate, in contrast to the universally applied distance-based relationship of Bedmap 1 and 2. They fill areas among Antarctica’s mountains that are largely unsurveyed, covering 757,960 cells or 8% of the data points used to interpolate the Bedmap3 thickness grid (and 0.6% of the total cells in this grid) (Table 4).

**Table 4:** Ice-thickness data types.

Thickness dataset	Points	%
Survey data	3,150,271	33.2%
Ice shelf: freeboard-derived	4,994,101	52.7%
Rock outcrops	299,252	3.2%
Synthetic: Glen flow law	757,960	8.0%
Synthetic: coastal margin	13,378	0.1%
Synthetic: streamlines	258,721	2.7%
*Total*	*9,473,683*	*100%*

These combined points represent 7% of the cells in the interpolated ice thickness grid. For the final thickness interpolation, note that we converted the streamlines described in Section *Synthetic ice thickness data: ‘streamlines’* to points with the attribute of linearly interpolated ice thickness, and distributed at the same 500 m spacing as the other point datasets.

#### Synthetic ice thickness data: grounded coastal margins

The grounded ice in contact with the ocean along Antarctica’s ice-shelf-free coasts is also little surveyed but its thickness can be constrained physically given that it: i) has known surface height above sea level (Section *Surface elevation*); ii) has a finite range of densities; iii) has known surface flow speed^33^; and iv) is subject to buoyancy but is not afloat (Section *Masks of grounded ice, transient grounded ice, floating ice shelf and rock*). To provide a thickness constraint for interpolation, we generated a perimeter of points along the shelf-free coastline at locations >2 km from survey data and >1 km from flow-law-derived data (see *Synthetic ice thickness data: Glen’s flow law for ‘thin ice’*). We assumed that relatively fast-flowing grounded coastal margins (>200 ma^−1^ ref. ^43^) are close to flotation and estimated their thickness as a factor of nine times the geoid-referenced surface height (after ref. ^41^). For slower-flowing grounded margins (50–200 ma^−1^), we assumed relatively well-grounded ice and assigned a thickness of twice the height (Section *Surface elevation*), and for the slowest-flowing areas (<50 ma^−1^) or those areas lacking surface flow speed data, we assigned a thickness equal to height. These 13,378 synthetic thickness points have a distribution skewed to low values, with a mean of 70 m (SD 80, median 51 m, IQR 75 m), and contribute 0.1% to the total (Table 4).

#### Synthetic ice thickness data: ‘streamlines’

**Fig. 6:**
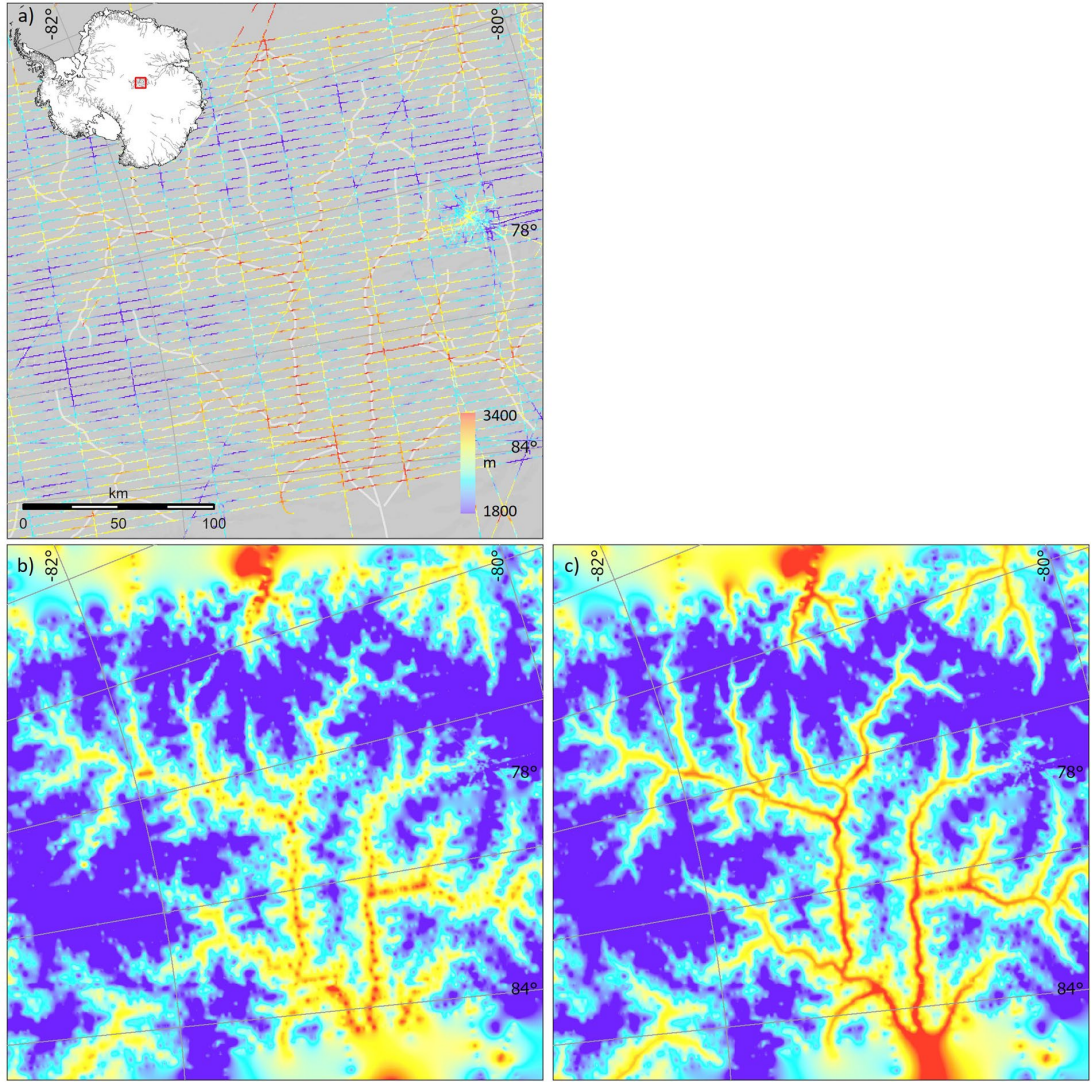
The depiction of subglacial troughs can be improved by identifying linear features common to multiple neighbouring survey lines. (**a**) A relatively dense grid of surveyed ice thickness (coloured lines) over the subglacial Gamburtsev Mountains overlain on digitised streamlines (white) that link points of local maximum thickness in neighbouring survey lines. Inset map shows the Antarctic-wide distribution of digitised streamlines in grey; (**b**) a grid interpolated using only surveyed thicknesses, where troughs appear ‘beaded’; (**c**) a grid interpolated using both surveyed thicknesses and thicknesses linearly interpolated along streamlines, resulting in smooth trough long profiles.

Elongated topographic troughs are common in glaciated landscapes^54^ but both survey patterns and survey-driven interpolations, as used here (Section *Interpolation of the ice thickness grid*), are typically not designed to follow subglacial troughs and so struggle to represent the continuity of these features in the Antarctic bed (Fig. 6). Trough continuity is often visually apparent from an aligned sequence of topographic lows in neighbouring surveyed cross profiles, which are often well captured by densely sampled measurements along flightlines (Fig. 6a). In some cases, this sequence of lows is replaced by an aligned sequence of survey-data gaps where the radar locally failed to detect the bed, which itself is useful evidence of a trough of relatively thick and warm-based ice^28,55^. The presence of troughs may also be supported by the alignment of exposed rock walls, visible ice-surface flowlines in satellite images or the surface DEM, or from bands of enhanced surface flow (e.g., refs. ^56–59^). Mass-continuity approaches to thickness interpolation (e.g., BedMachine^18^) effectively use bands of enhanced surface flow speed to guide ice thickness interpolation along troughs but require ice-flow assumptions that may preclude their use in other glaciological analyses^8^, and do not apply where the ice is thick but slow flowing and therefore largely decoupled from the detailed bed topography (e.g.^24^).

For Bedmap3, we employed a simple, linear interpolation approach to ensure that the long profiles of troughs are represented as continuous and smoothly varying bed features that span the often-large gaps between survey lines (Fig. 6). This approach expands upon that previously used by Bedmap2, which employed a set of eleven manually defined profiles of ice thickness along glacier centre lines that were linear interpolations between survey measurements at either end of each profile^8^. Here, we manually defined nearly 5000 along-trough ‘streamline’ segments that link sequential low points (trough bottoms) in adjacent pairs of surveyed cross profiles (Fig. 6a). We used these streamlines within the ArcGIS ‘Topo to Raster’ algorithm to enforce linear interpolation between these topographic lows (Section *Interpolation of the ice thickness grid*). We identified the course of trough long profiles primarily from alignments in survey data (which are particularly noticeable when the survey data are first gridded without the benefit of streamlines, e.g., Fig. 6b), and secondarily from their surface expression identified in the supporting datasets described above. Notably, though, not all troughs have a surface expression. In some cases, the survey data revealed troughs deviating from surface flowlines or continuing upstream into the slow-flowing ice sheet interior. Our guided, survey-driven interpolation therefore allows us to map such troughs that are not amenable to mass-conservation approaches guided by surface flow. We also employed streamlines to enforce linear interpolation of thickness along a small number of ice divides and margins where unguided interpolation performed poorly (see Data Records: bm3_streamlines_pt).

#### Interpolation of the ice thickness grid

**Fig. 7:**
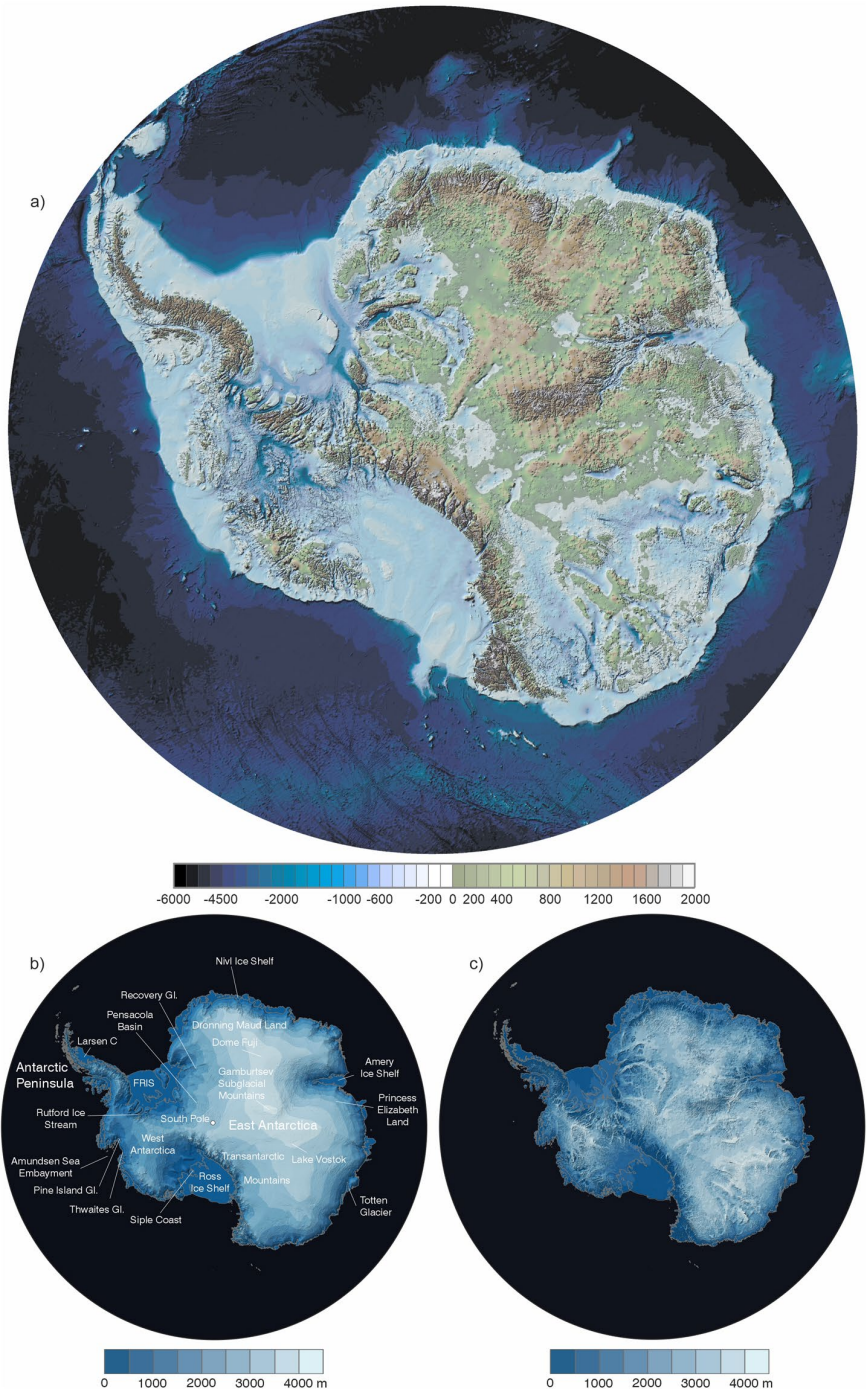
Bedmap3 grids of (**a**) bed topography and (**b**) surface elevation, in metres above sea level (g104c geoid), and (**c**) ice thickness in metres. Locations labelled in (**b**) are referred to in the text.

To produce a continuous grid of ice thickness^45^ (Fig. 7c, Data Records) from the summarised survey data points (Section *Filtering and weighting of survey data*), derived ice shelf thickness (Section *Ice shelf thickness*), synthetic thicknesses (Sections *Synthetic ice thickness data: Glen’s flow law for ‘thin ice’, Synthetic ice thickness data: grounded coastal margins*, *Synthetic ice thickness data: ‘streamlines’*) and rock outcrops (Table 4) we used the Topo-to-Raster (formerly Topogrid) ArcGIS algorithm previously employed in Bedmap2, with the same gridding options (no drainage enforcement, data type “spot”, maximum iterations 20, roughness penalty 0.5, profile curvature roughness penalty 0, discretisation error factor 1 (after ref. ^8^)). This algorithm is based on adapted thin plate splines with an iterative finite difference interpolation^60,61^ and has proved well suited to representing glaciated landscapes^8^. It fits a series of locally varying curved surfaces to all the data points while seeking to minimise the curvature, according to a specified roughness penalty. As such, it inherently produces a representation of a surface that conforms closely to data where available and is smoothly continuous throughout, but it also allows deviations to these curved surfaces to be imposed through, for example, streamlines. This approach is particularly valuable in ensuring seamless, splined transitions across gaps between the disparate ice thickness data sources described above, including the transition from grounded ice to ice shelf, while also interpolating linearly along the trough long profiles that we define.

### Surface elevation

To represent the surface elevation of the Antarctic ice sheets, shelves and rock^45^ (Data Records, Fig. 7b), we used a recently published, gap-filled version of the REMA Digital Elevation Model that was constructed with high absolute accuracy (~1 m) using high-resolution optical imagery (~10 m) spanning 2007-2017 (mean 2015) and covering 95% of continental Antarctica^62^. The ‘Gapless-REMA100’ version used here completes the continental coverage by filling voids with a range of other elevation data and interpolation, plus resampling to a 100 m grid^41^.

Along some coastal margins we manually removed elevation anomalies in Gapless-REMA100 consisting of apparent high-elevation spikes that are not supported by satellite images of the landscape (possibly due to coastal clouds), or near-zero-elevation coastal lows that appear to represent sea areas misclassified as ice shelf. Ice shelf front locations change continuously, and where they differed between Gapless-REMA100 and Bedmap3, introducing data gaps in the Bedmap3 surface, we assigned these gap cells the mean elevation of the neighbouring mapped cells, calculated over radii ranging from 5-50 cells, according to the size of the gap. We also applied a median filter of radius 3 to 5 cells to the surface elevations to remove small-scale localised anomalies, including ice shelf crevasses that were mapped by REMA but are not captured by the Bedmap3 thickness data. We then resampled and reprojected the elevation grid to the standard 500 m Bedmap3 grid and referenced the vertical elevations to mean sea level by converting from the Gapless-REMA100 WGS84 datum to the g104c geoid, as used by Bedmap2. To ensure consistency with the Bedmap3 rock and ice masks, we reset any grid cells with negative or zero elevations relative to sea level to + 1 m.

### Bed topography

To generate a bed topography for the grounded ice sheets^45^ (Fig. 1, Data Records), we subtracted our ice thickness grid (Section *Interpolation of the ice thickness grid*) from our surface topography grid (Section *Surface elevation*). Within the resulting bed grid, we fixed rock outcrop elevations (with zero ice thickness) to equal the surface elevation, and we additionally subtracted the water column thickness of subglacial lake Vostok, as for Bedmap2 (ref. ^8^). We then combined this grid of the grounded ice sheet bed with the recently published IBCSO v2 bathymetric grid of the Southern Ocean^63^ that we updated with more recent regional grids in areas near the Nivl^64^ and Totten^65^ ice shelves, to create a seamless ice bed and seafloor grid at 500 m spacing from 60 °S to 90 °S (Fig. 7a).

The process of combining the grounded bed and bathymetry grids must be done with care because the dynamics of ice flow in the grounding zone where these grids meet are critically sensitive to local bed topography, ice thickness and water depth. Changes in these ice dynamics are fundamental to modelled projections of ice sheet and sea level change^11^ hence artefacts in gridded bed elevation, such as steps between mismatched bathymetry and bed grids, or unphysical combinations of gridded surface height, ice thickness and bed, could introduce significant errors in modelled ice sheet behaviour.

As Antarctica’s coasts and sub-ice-shelf cavities remain among the least-well surveyed areas of the global ocean, with most ice shelves having no direct measurements of bathymetry^28^, further interpolation was needed. No straightforward, universal approach to interpolation of the bed through the grounding zone performs well in all settings because the form and complexity of coastlines and the sub-shelf grounding line vary over a wide range of scales, from the deeply indented, steep sided fjords of the Antarctic Peninsula to the smooth, broad, and gentle submarine slopes of the Siple Coast (Fig. 7b). However, certain logical constraints to bed elevation can be applied: for grounded ice at the coast, the known surface elevation and ice thickness must combine with a bed elevation consistent at every cell with the ice not being afloat. Conversely, every cell of floating ice must be thin enough and low enough to float and have a positive water column thickness below it, defining an upper limit to the bed elevation. In the absence of bathymetric survey data, we also assume that the form of the bed in a zone seaward of the grounding line or open coastline is smoothly continuous with the surveyed bed upstream and downstream, on the grounds that landscapes either side of the contemporary grounding line have a similar glacial-geomorphological history (e.g.^66^).

To minimise artefacts when joining the grounded bed and coastal bathymetry we employed a tailored approach, iterated several times. This eliminated unphysical configurations of ice, water and bed that do not agree with their known floating/grounded status and avoided introducing abrupt steps in the submarine landscape. To ensure a smooth transition, we cropped out the bathymetry grid (less well surveyed than the grounded ice) by 10 km seaward of the marine grounding line or open coastline (Section *Masks of grounded ice, transient grounded ice, floating ice shelf and rock*), and lakeward of the Lake Vostok grounding line, with cropping distance subsequently modified as needed to avoid visible artefacts in the resulting merged bed. We then interpolated across this ≥ 10 km wide coastal gap between the 500 m-spaced, interpolated elevation points from the bathymetry and grounded bed grids, as in Section *Interpolation of the ice thickness grid*.

To correct logical errors in our interpolated bathymetry (see Technical Validation), we lowered the seabed where necessary to ensure a negative elevation relative to sea level and a positive water column thickness for all ungrounded cells at all stages of the tide (with integer tidal ranges from 1 to 7 m, from the FES2014 tidal model^67^). In the sub-ice-shelf cavities, we enforced a positive water column thickness below the free-floating ice shelf base of greater than the maximum tidal range^67^ for each cell, a value that implies flotation throughout the tidal cycle. For ice shelf areas identified as ‘transient grounded’ (Section *Masks of grounded ice, transient grounded ice, floating ice shelf and rock*), we enforced a water column thickness of specifically half of the maximum tidal range (integer from 1 to 4 m), consistent with flotation with transient grounding at low tide.

### Bedmap3 timestamps

The Bedmap3 components are current for the approximate period 2007-2022. The outer coastline dates from 2022 and the grounding line from ~2015-2020. The mean survey year for the thickness survey dataset is 2006.9 (SD 7.6 years), somewhat more recent for areas of extensive recent survey such as the main lower trunk of Pine Island Glacier (2009.4, SD 5.4 years) and Thwaites Glacier (2010.6, SD 5.7 years) (Table 2). Ice shelf thickness was derived from 2011–2014 altimetry, surface topography from 2007-2017 and bathymetry from the period up to 2020.

### Comparison to earlier Bedmap products and BedMachine v3

**Fig. 8:**
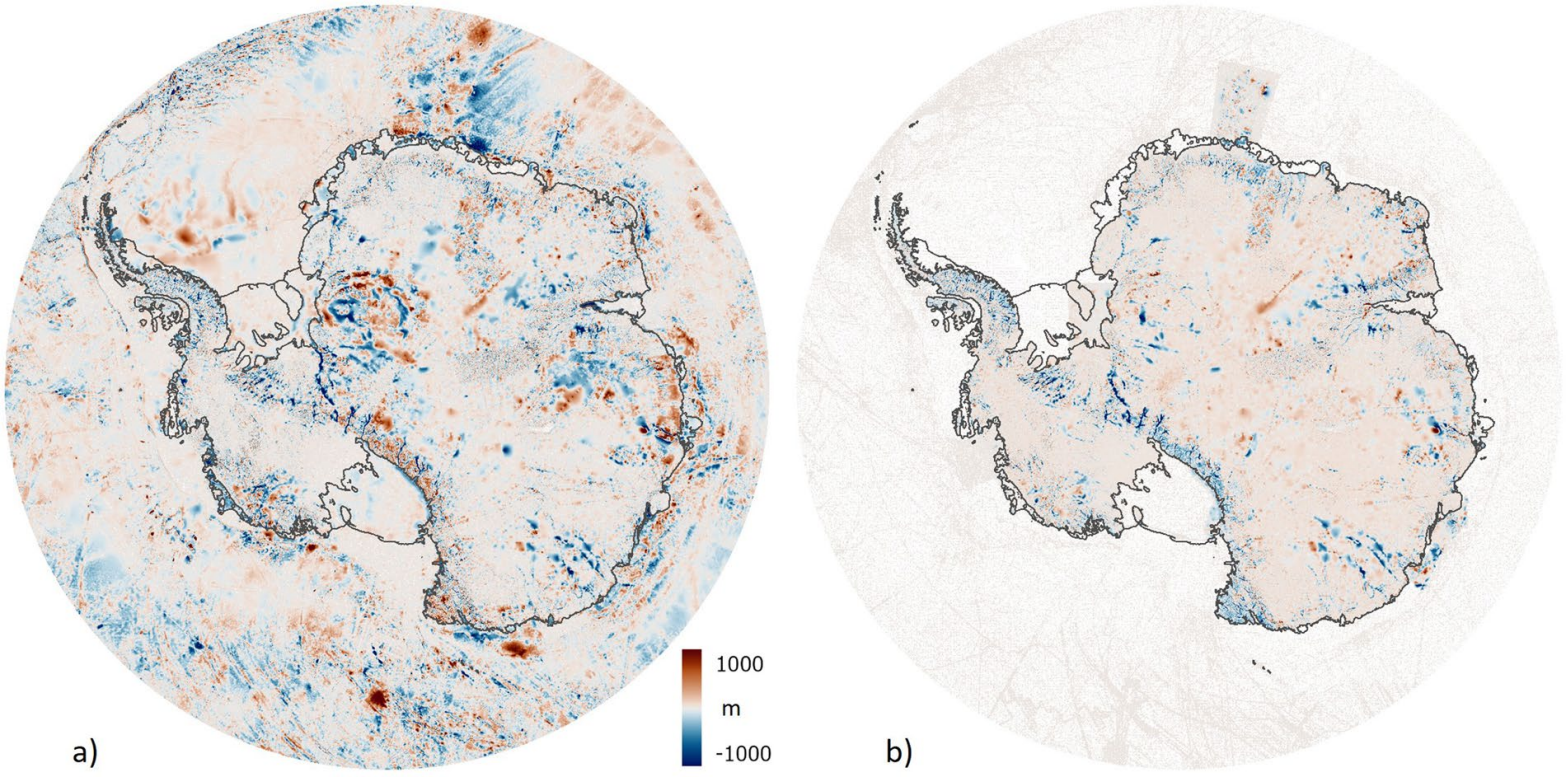
The Bedmap3 bed elevations can be compared to earlier products. (**a**) Bedmap3 minus Bedmap2; (**b**) Bedmap3 minus BedMachine v3 (red indicates that the Bedmap3 bed is higher, blue that it is lower).

**Fig. 9:**
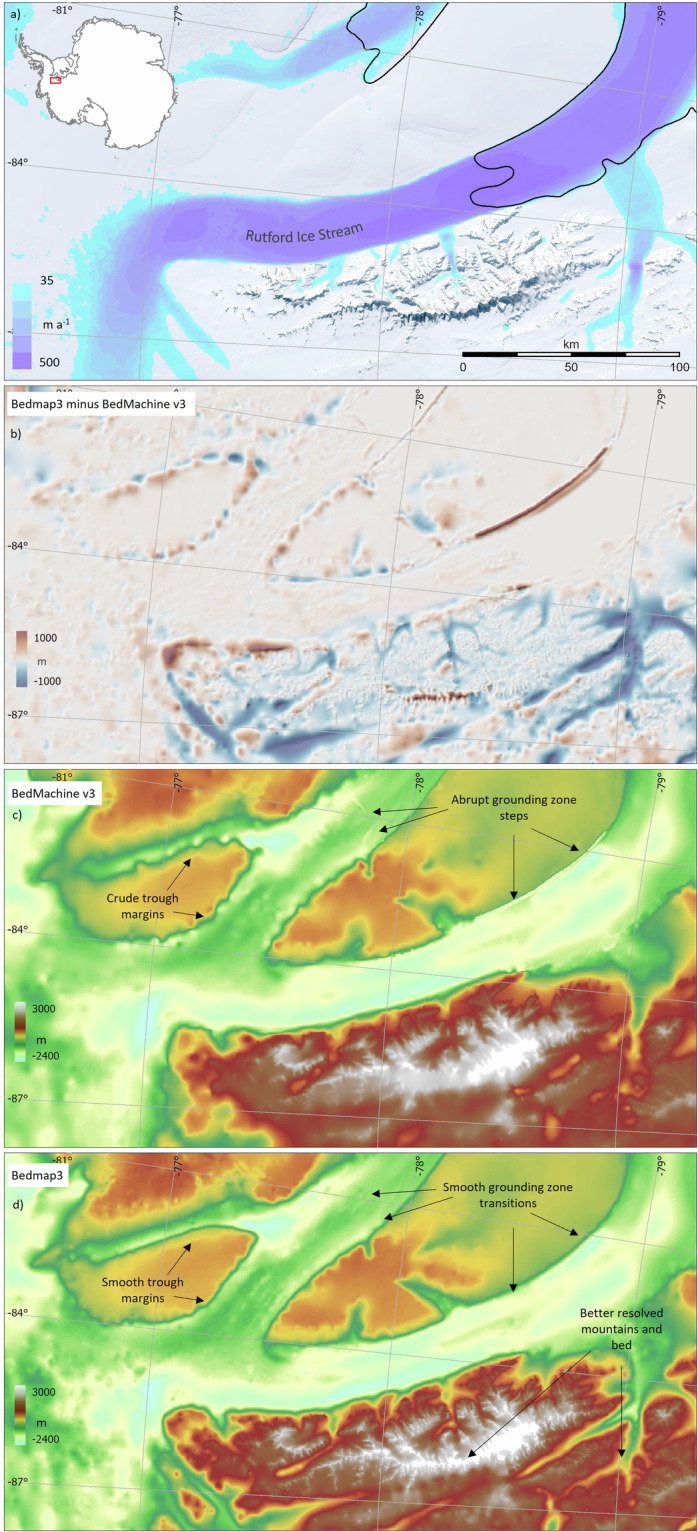
The Bedmap3 bed has some subjective advantages over BedMachine v3, including smoothly continuous grounding zones and troughs, and better-resolved areas of bed. (**a**) surface flow speed^43^ and Bedmap3 grounding line (solid black) in the Rutford Ice Stream area; (**b**) map of Bedmap3 minus BedMachine v3 bed topography; (**c**) BedMachine v3 bed topography; and (**d**) Bedmap3 bed topography, highlighting areas of difference. Similar BedMachine v3 issues have been reported previously in this area^71^.

**Fig. 10:**
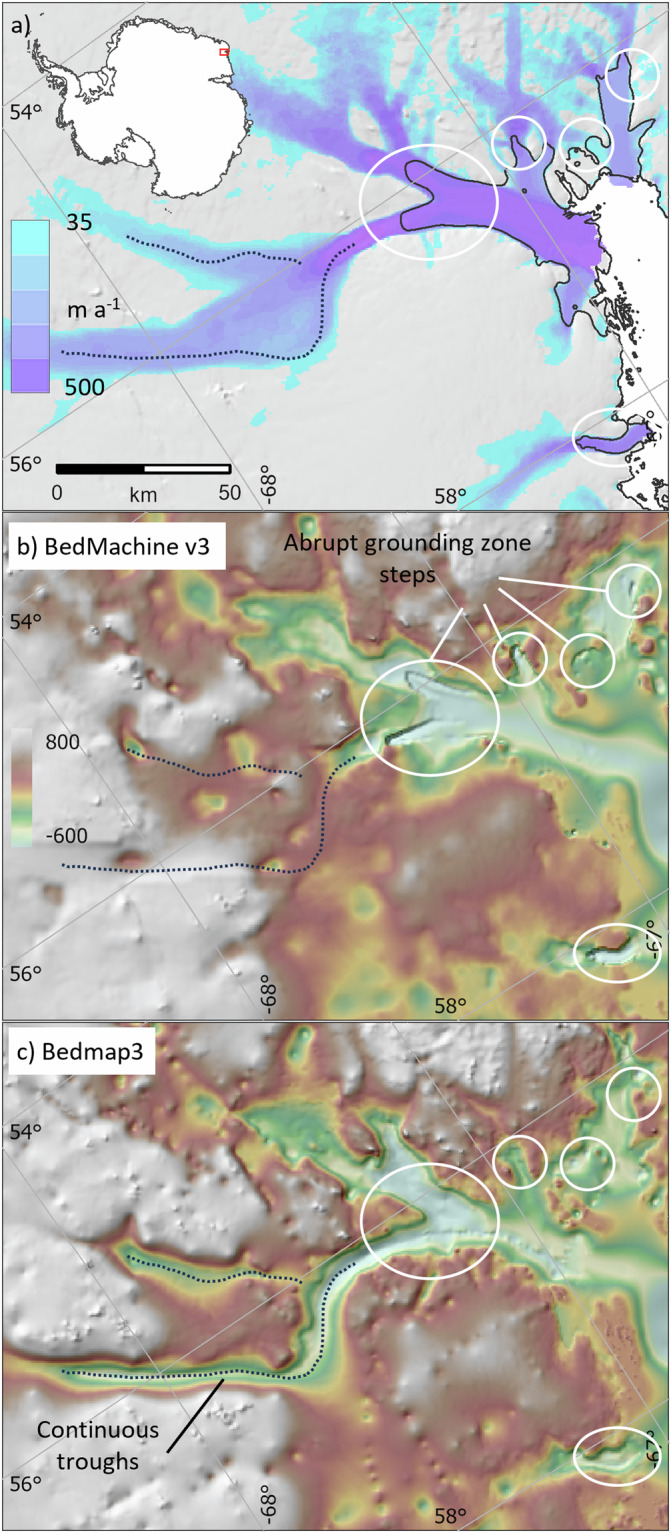
Bedmap3 features smoothly continuous grounding zones and troughs, in contrast to some sites in BedMachine v3. (**a**) Surface flow speed^43^ and Bedmap3 grounding line (solid black) in the Wilma Glacier region; (**b**) BedMachine v3 bed topography; (**c**) Bedmap3 bed topography, both overlaid on shaded relief. The dotted line highlights troughs that are continuous in Bedmap3, white ovals highlight abrupt steps in the BedMachine v3 grounding zone.

**Fig. 11:**
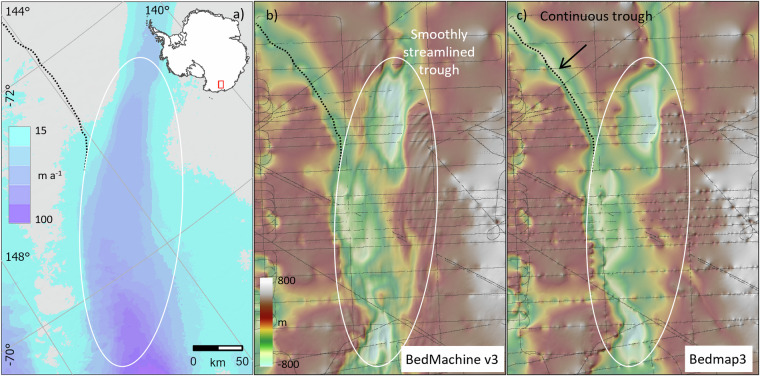
The BedMachine v3 bed has some subjective advantages over Bedmap3 where glacier flow is relatively fast, but survey data are sparse. (**a**) Surface flow speed^43^; (**b**) BedMachine v3 bed topography; (**c**) Bedmap3 bed topography, both overlaid on shaded relief and with survey data shown as grey lines. The black dotted line highlights a trough under slow-flowing ice that is more smoothly continuous in Bedmap3. The white oval highlights the trough of a relatively fast-flowing ice stream that in BedMachine v3 is more smoothly streamlined and subjectively more realistic than in Bedmap3, given the typical streamlined form of deglaciated ice stream landscapes (e.g., ref. ^54^). An objective test of bed accuracy in this area requires more survey data.

Relative to Bedmap2, there are large and widespread changes in the Bedmap3 bed topography (Fig. 8a, Table 5). The contrast with BedMachine v3 is less marked on the continental scale (Fig. 8b, Table 5) but, as a result both of new survey data and the difference in approach to interpolation and synthetic data, Bedmap3 includes hundreds of troughs under the grounded ice sheet that are more clearly defined, plus substantial areas with more highly resolved bed and mountain features (e.g., Fig. 9). Importantly, Bedmap3 has multiple transitions that are smooth rather than abrupt through the grounding zone where the bathymetry and grounded-ice grids meet (e.g., Figs. 9, 10). For some areas of fast flow along ice stream troughs, however, particularly where surveys are relatively sparse, the BedMachine mass conservation approach to interpolation yields a more smoothly continuous and subjectively plausible trough form (e.g., Fig. 11). In the absence of survey campaigns that achieve systematic sampling of ice thickness at resolutions approaching the 500 m grid spacing used here, we therefore suggest that the most accurate representation of the grounded Antarctic ice sheet bed would likely result from a combination of the mass-conservation and Bedmap3 approaches, taking advantage of their respective strengths. Such a combination would, however, come with the caveat that the bed topography would locally have some dependency on the flow-modelling assumptions employed in mass conservation.

**Table 5:** Statistical comparison of Bedmap1, Bedmap2, BedMachine v3 (BMv3) and Bedmap3.

	Bedmap1	Bedmap2	BMv3	Bedmap3
Area including ice shelves (10^6^ km^2^)	13.99	13.92	13.59	13.63
Area excluding ice shelves (10^6^ km^2^)	12.35	12.30	12.35	12.10
Volume including ice shelves (10^6^ km^3^)	26.07	26.92	26.77	27.17
Volume excluding ice shelves (10^6^ km^3^)	25.34	26.54	26.06	26.42
Mean thickness including ice shelves (m)	1859	1937	1953	1948
Mean thickness excluding ice shelves (m)	2034	2126	2118	2148
Thickest ice (m)*	4897	4897	4822	4757
Mean elevation of the grounded bed** (m)	155	83	72	74
Deepest bed point below sea level** (m) (Byrd Glacier)	−2496	−2870	−3827***	−2973
Area below sea level** (10^6^ km^2^)	5.01	5.50	5.60	5.65
% of grounded area lying below sea level**	40.6	44.7	45.3	46.7
Potential sea-level equivalent (m)	57	58	58	58

*This location was previously Astrolabe Basin, but in Bedmap3 is an un-named canyon at 76.052°S, 118.378°E. **Relative to the gl04c geoid. ***BedMachine V3 modelled deepest bed depth, West Lambert Glacier.

## Data Records

The surface, thickness, bed, mask and uncertainty grids, and a gridded count of survey data points, are available from 10.5285/2d0e4791-8e20-46a3-80e4-f5f6716025d2 in 16-bit signed integer tiff and netcdf formats, at 500 m spacing with 13334 columns and 13334 rows, and a no-data value of -9999 (ref. ^45^). Elevation and uncertainty units are metres in all cases. The grid names are: bm3_bed; bm3_surface; bm3_thickness; bm3_bed_uncertainty; bm3_thickness_uncertainty; bm3_masks; bm3_thickness_survey_count. Also available at this site is a point shapefile set of the linearly interpolated streamline thicknesses, called bm3_streamlines_pt.

## Technical Validation

### Quality control checks

We applied a set of quality-control checks and corrections to all grid cells within the final suite of Bedmap3 grids that consisted of:

- All ‘sea’ cells have negative bed height relative to sea level.
- All ‘non-sea’ cells (grounded ice, rock, floating or transiently grounded ice shelf) have positive surface height relative to sea level.
- All ‘grounded ice’ cells have positive surface height and ice thickness.
- All ice shelf cavity cells have a positive water column thickness.
- Surface-minus-thickness-minus-bed = 0 for all grounded cells.
- All transient-grounded ice shelf cells have column thickness = (tidal range/2), with a minimum cavity of 1 m.
- All rock cells have thickness = 0 and positive surface and bed height.
- Grid extents are consistent, and grid cells align exactly.
- Data type (integer) is consistent throughout.
- Mask cell categories agree with visible satellite imagery (1 = grounded ice, 2 = transiently grounded ice shelf, 3 = floating ice shelf, 4 = rock).
- No sharp discontinuities exist in ice thickness at the boundaries between masks.
- No sharp discontinuities exist in the bed at boundaries between masks or elsewhere.
- No grounded coastal ice cells have surface height and thickness that would imply flotation (thickness should not exceed 10 × height) where in contact with the sea.

### Uncertainty estimates

We estimate uncertainty in the Bedmap3 grids through error budgets based upon a combination of published uncertainties and our own tests of accuracy and precision. The input datasets inherently have uncertainties with differing statistical distributions, and not all are well known. Furthermore, most Bedmap3 cells have values that are interpolated between sparse observations, and the uncertainties in this process are also difficult to quantify. Our uncertainties are therefore a best estimate based on the available evidence, given to approximately the 1-sigma (SD) level.

#### Surface DEM uncertainty

Relative to other altimetry products, the REMA DEM has a bias close to zero and a Root Mean Squared Error (RMSE) uncertainty of < ± 1 m for most of Antarctica, and < ± 2.4 m over steeper slopes in mountain ranges and some coastal areas^62^. Assessed similarly, the gapless 100 m gridded version of REMA used here has relative bias over the filled voids averaging <1 m and RMSE uncertainty of ±3 m on average^41^. In the absence of ground control, however, the absolute vertical accuracy of the REMA DEM is estimated as ±4 m (https://www.pgc.umn.edu/guides/stereo-derived-elevation-models/pgc-dem-products-arcticdem-rema-and-earthdem/, accessed 3 July 2024). We therefore estimate the combined average Bedmap3 surface elevation uncertainty as ±7 m. We note, however, that void filling produced a maximum RMSE of ±14 m for an area covering 0.06% of the Antarctic Peninsula^41^, which suggests local absolute elevation errors up to ±18 m.

#### Ice thickness uncertainty

Ice thickness surveys have individual measurement uncertainties, and per-cell summary statistics have additional sampling uncertainties. Evidence for individual measurement precision comes from crossover analysis of co-located thickness measurements. The differences found at 600,973 crossovers had occasional large outliers (hundreds of metres) associated with navigation errors in early measurements, but a (non-Gaussian) distribution with an interquartile range (IQR) of 5 m (ref. ^8^). The 3,261,006 statistically summarised 500 m cells of surveyed thickness for the Bedmap3 grounded ice sheet have a mean of 17.7 individual measurements per cell^17^, with a thickness standard error (SE) of 3.6 m for cells with multiple measurements, indicative of sampling uncertainty. These tests suggest that surveyed cells typically have a combined 1-sigma thickness uncertainty of < ± 10 m for most summarised cells, and an estimated ±20 m for ~800,000 cells with only a single measurement (up to hundreds of metres in extreme cases). Rock outcrops additionally provide a zero-thickness constraint that is mapped with high resolution, confidence, and accuracy (Section *Masks of grounded ice, transient grounded ice, floating ice shelf and rock*). When surveyed cells are represented in an interpolated grid their values can be modified by the spline-fitting interpolation process. However, after interpolation, we reset thicknesses to zero at rock outcrops, and for surveyed ice-thickness cells the effect of interpolation is small, with a mean difference between the measured and interpolated thickness for the same cell of 0.3 m (SD = 41, median = 0 m, IQR = 6 m)).

Our synthetic thickness datasets have greater uncertainties. In a test of our flow-law-derived ‘thin ice’ thicknesses around rock outcrops against 84,984 observations, we found a non-Gaussian distribution with IQR = 152 m (see *Synthetic ice thickness data: Glen’s flow law for ‘thin ice’*). The uncertainty in our synthetic coastline thicknesses (Section *Synthetic ice thickness data: grounded coastal margins*) are difficult to quantify directly but as they are relatively small (mean thickness of 70 m) and are constrained by surface elevations (Section *Surface elevation*), we estimate their uncertainty as ± 15 m (~20% of the mean).

Ice-shelf thickness bias arising from the DEM used to determine the freeboard above flotation was previously found to be < 1 m, which translates into a < 9 m thin bias^47^. We corrected for such bias by calibrating to survey data with typically metres-scale thickness corrections (see *Ice shelf thickness*; Table 3). Survey calibration data are limited in extent and may have their own biases and uncertainties, however. On the FRIS, for example, we found a mean ice thickness difference of 4.8 m between survey data from radar (n = 396) and seismics (n = 529) that lie within 2 km of each other (and fall outside of marine-ice areas), and the radar crossover analysis described above suggests metres-scale precision for these survey data. Further shelf thickness uncertainty arises from estimates of the column-averaged density (order metre scale) and the failure of hydrostatic equilibrium (potentially order 100 m). We therefore estimate a residual uncertainty in ice shelf thicknesses of typically ± 10 m.

Away from ice thickness observations and constraints such as rock outcrops, thickness is interpolated for 93% of Bedmap3 grid cells. We assessed interpolation error by comparing thickness observations new to Bedmap3 to the interpolated Bedmap2 thickness grid, which employed the same interpolation algorithm but without these new data. For 728,951 new cells lying more than 500 m from Bedmap2 survey points, the differences have a Gaussian distribution with a mean of 17 m and a Mean Absolute Difference of 171 m (IQR 188 m), indicative of the magnitude of the interpolation error. Spatially, we found that absolute difference, *y*, increases logarithmically with distance from data, *x*, according to,

2 $${\rm{y}}=38.479\mathrm{ln}({\rm{x}})-166.11$$

The dependence of interpolation error on distance (x) from data.

**Fig. 12:**
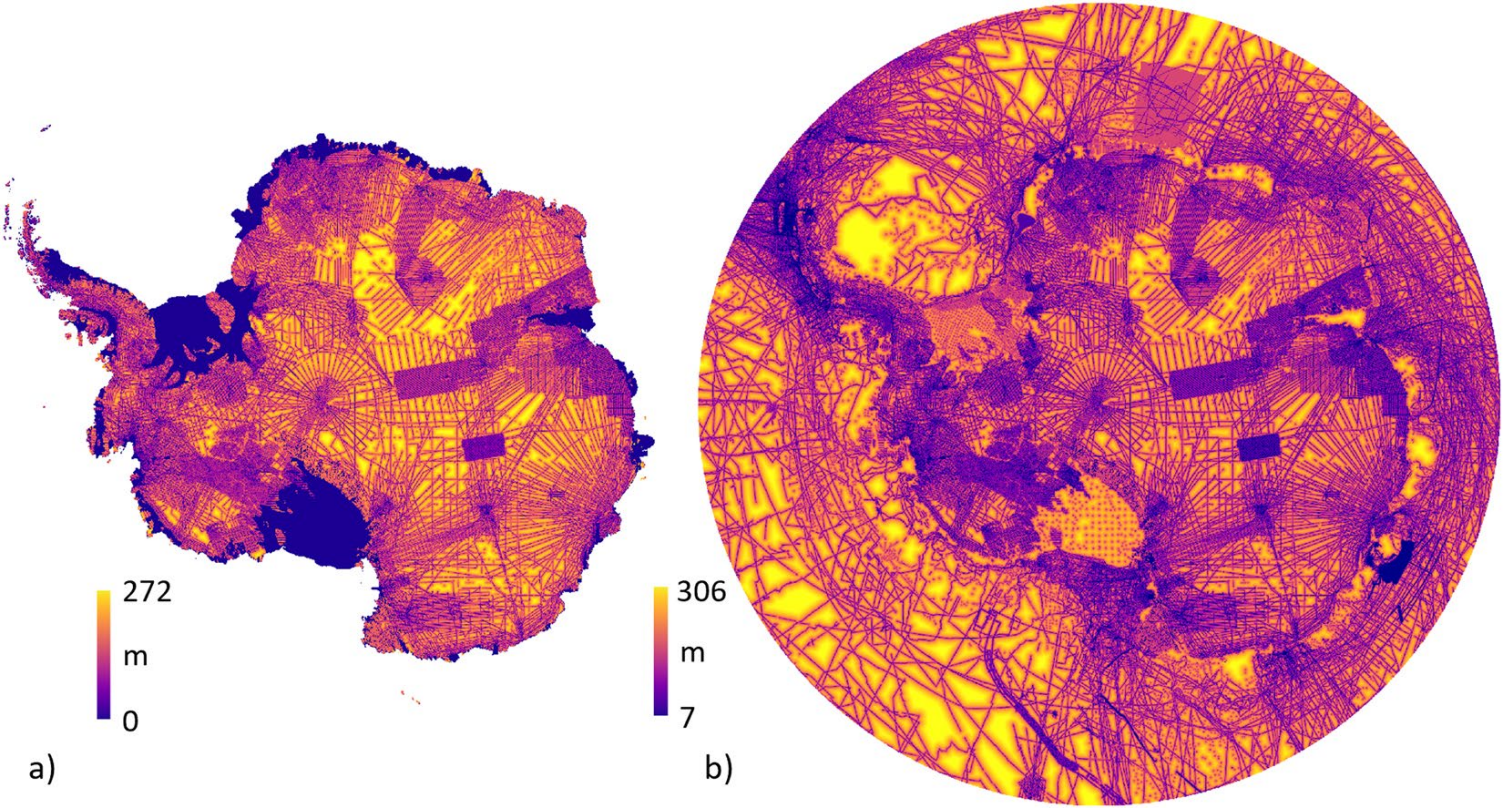
Estimated 1-sigma uncertainty map for (**a**) ice thickness and (**b**) bed topography. Zero thickness uncertainty in (**a**) applies to rock areas.

We used this relationship to map interpolation uncertainty from a minimum of ±73 m at 500 m distance from data to a maximum ±272 m at 98 km distance (and ±166 m at the average distance of 5.6 km) (Fig. 2, Fig. 12a). We combined these classes of uncertainty into one ice thickness uncertainty grid^45^.

#### Bed topography uncertainty

Uncertainty in the elevation of the grounded bed^45^ comes from the uncertainty in the grids of surface topography and ice thickness (Table 6) combined in quadrature, which suggests a 1-sigma absolute uncertainty ranging from ±7 m (rock) to ±12 m (surveyed) and ±276 m (interpolated). Uncertainty in IBCSO v2 bathymetry is not reported^63^ though it is largely based upon single-beam and multibeam echo sounding surveys covering 24% of Southern Ocean cells^63^ with a sounder vertical precision of 0.1 to 50 m and quoted instrument accuracy of 0.2% of depth^63^, equivalent to <10 m in most cases. The remaining 76% of the bathymetry was interpolated, however, and even on the relatively shallow continental shelf (~500 m depth), independent estimates of IBCSO v2 accuracy suggest a shallow bias averaging at least 224 m (and locally exceeding 1000 m) for a sample of 9787 grid cells of size 10 × 10 km (ref. ^64^). The independent mapping used here to update bathymetry under the Nivl Ice Shelf, adjacent continental shelf and farther offshore found IBCSO v2 shallow biases of 168 m, 19 m, and 7 m respectively, with extreme differences of ±500 m. Standard deviations of these differences were 180 m, 128 m and 115 m respectively, and uncertainty in the revised grid for this area was estimated as ±138 m to ±160 m (uncertainties that are relatively large as this mapping was based on inversion of gravimetry data)^68^. Similar bathymetry bias of up to 250 m was reported for the interpolated IBCSO v1 grid relative to more recent sounding data offshore of Totten Glacier^65^. We incorporated these more recent regional grids into the Bedmap3 bathymetry, but similar biases to these identified locally are likely to be present elsewhere on the continental shelf where former glacial troughs are not adequately sampled by bathymetric surveys. These findings suggest 1-sigma absolute uncertainties in bathymetry ranging from around ±10 m (surveyed) to ±300 m (interpolated), similar to those for the grounded ice sheet bed. Given these similarities, we estimated the uncertainty in interpolated bathymetry as a function of distance from data in the same way as for the grounded ice sheet thickness (Equation (2)). This yielded an interpolation uncertainty range of ±60 m at the minimum 500 m distance to ±306 m at the maximum distance of 211 km, which we map with the estimated ±10 m uncertainty for surveyed cells (Fig. 12b).

**Table 6:** Summary of Bedmap3 uncertainties.

Class	Uncertainty (±m)
*Ice thickness*	
Surveyed cells	10
Surveyed cells (single measurements)	20
Rock cells	0
Synthetic ‘thin ice’ cells	152
Synthetic coastal cells	15
Interpolated cells	73 to 272
Ice shelf	10
*Elevation*	
Surface DEM (including rock)	7
Grounded bed	7 to 276
Surveyed seabed	10
Interpolated seabed	60 to 306

## Data Availability

No custom code was used in producing these datasets.
